# Bias in medical AI: Implications for clinical decision-making

**DOI:** 10.1371/journal.pdig.0000651

**Published:** 2024-11-07

**Authors:** James L. Cross, Michael A. Choma, John A. Onofrey

**Affiliations:** 1 Yale School of Medicine, New Haven, Connecticut, United States of America; 2 Department of Radiology & Biomedical Imaging, Yale University, New Haven, Connecticut, United States of America; 3 Department of Urology, Yale University, New Haven, Connecticut, United States of America; 4 Department of Biomedical Engineering, Yale University, New Haven, Connecticut, United States of America; Mayo Clinic Rochester: Mayo Clinic Minnesota, UNITED STATES OF AMERICA

## Abstract

Biases in medical artificial intelligence (AI) arise and compound throughout the AI lifecycle. These biases can have significant clinical consequences, especially in applications that involve clinical decision-making. Left unaddressed, biased medical AI can lead to substandard clinical decisions and the perpetuation and exacerbation of longstanding healthcare disparities. We discuss potential biases that can arise at different stages in the AI development pipeline and how they can affect AI algorithms and clinical decision-making. Bias can occur in data features and labels, model development and evaluation, deployment, and publication. Insufficient sample sizes for certain patient groups can result in suboptimal performance, algorithm underestimation, and clinically unmeaningful predictions. Missing patient findings can also produce biased model behavior, including capturable but nonrandomly missing data, such as diagnosis codes, and data that is not usually or not easily captured, such as social determinants of health. Expertly annotated labels used to train supervised learning models may reflect implicit cognitive biases or substandard care practices. Overreliance on performance metrics during model development may obscure bias and diminish a model’s clinical utility. When applied to data outside the training cohort, model performance can deteriorate from previous validation and can do so differentially across subgroups. How end users interact with deployed solutions can introduce bias. Finally, where models are developed and published, and by whom, impacts the trajectories and priorities of future medical AI development. Solutions to mitigate bias must be implemented with care, which include the collection of large and diverse data sets, statistical debiasing methods, thorough model evaluation, emphasis on model interpretability, and standardized bias reporting and transparency requirements. Prior to real-world implementation in clinical settings, rigorous validation through clinical trials is critical to demonstrate unbiased application. Addressing biases across model development stages is crucial for ensuring all patients benefit equitably from the future of medical AI.

## Author summary

In this work, we explore the challenges of biases that emerge in medical artificial intelligence (AI). These biases, if not adequately addressed, can lead to poor clinical decisions and worsen existing healthcare inequalities by influencing an AI’s decisions in ways that disadvantage some patient groups over others. We discuss several stages in the process of developing a medical AI model where bias can emerge, including collecting data, training a model, and real-world application. For instance, the way data is collected can exclude or misrepresent certain patient populations, leading to less effective and inequitable AI systems. We provide examples, both hypothetical and real, to illustrate how these biases can alter clinical outcomes. These examples show that biases are not just possible; they are a significant risk if not actively countered. Our review stresses the importance of diverse and comprehensive data sets, sophisticated statistical methods to remove biases, and clear reporting standards—key components of a future where medical AI works equitably and supports high-quality clinical care for everyone.

## Introduction

The application of artificial intelligence (AI) algorithms to the medical domain has exploded in recent years, facilitating tremendous advances in clinical tasks like risk prediction and disease screening [1,2]. These medical AI models are increasingly implemented in real-world settings for clinical decision support, providing early warnings, facilitating diagnosis, predicting treatment responses, and more [3–6]. An idealized promise of medical AI is its objectivity and reproducibility, removing provider biases and clinical inequities toward optimized and personalized care for all patients. In reality, however, AI models are frequently found to be biased toward certain patient groups, leading to disparities not only in performance but also in potential or actual clinical benefits [7–9].

Awareness of bias and the issues it poses—clinical, ethical, financial—is gradually increasing, as evident by the FDA’s recent Action Plan that emphasized the importance of mitigating bias in medical AI systems [10,11]. However, while bias detection and mitigation methods are relatively well developed for traditional statistical techniques like regression, the literature surrounding similar methods for AI algorithms is still nascent [12,13]. Additionally, there is a lack of standardized bias reporting guidelines within and across academic journals [12].

Here, we highlight how biases in medical AI—especially in applications that involve clinical decisions—occur and compound throughout the AI development pipeline. These biases, if left unchecked, will contribute to and exacerbate longstanding health disparities and negatively affect clinical care and decision-making. Methods to address these biases will prove crucial for ensuring the equitable realization of the current and future benefits of medical AI.

## Related work

The topic of bias in AI has garnered significant attention in recent years, with multiple review papers addressing different aspects of this critical issue [14–18]. Fewer studies have concentrated specifically on the implications and consequences of biased AI in healthcare [19–22]. While these reviews have made valuable contributions to our understanding of bias in medical AI, they differ in scope, depth, and focus, leaving a gap in understanding how bias permeates the development of AI for clinical decisions. Our work builds upon and extends these existing studies to provide a comprehensive and accessible overview of bias and its consequences in clinical decision-making.

Our approach distinguishes itself through several key aspects. While many reviews on bias focus on data and model development, we provide an extensive overview of bias across the entire AI development pipeline, from data collection to publication and real-world deployment. This approach allows us to examine how biases can compound and interact at different stages of model development. While some reviews concentrate on a single medical domain, particularly in medical imaging [23,24], our discussions integrate real-world examples across diverse medical domains, enabling unique analysis of cross-domain challenges and broader systemic issues, such as biases in funding and publication. Several reviews focus primarily on statistical biases present in AI models—particularly those arising from training data—along with corresponding statistical debiasing methods [17,25]. While these technical biases are crucial, our study includes extensive discussion of non-technical biases and their clinical consequences. We explore practical mitigations strategies for these biases in diverse healthcare settings, an area often underexplored in other works. Furthermore, we incorporate a section on recent advances in medical large language models (LLMs), examining their unique potential biases in medical applications.

Our review is presented in a narrative format, designed to be accessible to a broad audience, including healthcare professionals without a technical background in AI. This approach ensures that the insights we provide are not only theoretically robust but also practically useful. By addressing the gaps in the current literature and offering a comprehensive, up-to-date analysis, our review serves as an accessible resource for AI developers and healthcare professionals alike. It aims to enhance understanding of bias in medical AI and provides actionable guidance on how to mitigate its effects in downstream clinical decision-making.

## Artificial intelligence in medicine

AI seeks to develop algorithms and machines that exhibit human-like intelligence and cognition [26]. While traditional programming software rely on predefined and fixed instructions and rules, AI systems leverage huge swaths of data and mathematical models to learn patterns and make decisions based on the data they are exposed to [27]. The most popular subfield of AI is machine learning (ML), whose models learn through supervised (trained on data with “ground-truth” labels) or unsupervised (identifying patterns within data without prior knowledge of outcomes) paradigms [28].

In the healthcare domain, medical AI and ML models have demonstrated the potential to improve patient outcomes, reduce cost burdens, accelerate clinical trials, and revolutionize the way we diagnose, treat, and prevent disease [26,28–31]. State-of-the-art deep learning methods, which consist of computational models composed of multiple processing layers that can learn complex representations of data, have achieved unprecedented performance in imaging-based diagnosis, drug discovery, gene analysis, and natural understanding of medical language [32–37].

## Defining bias in medical AI

In the context of medical AI for clinical decision-making, we define bias as any instance, factor, or prejudice that drives an AI algorithm to produce differential or inequitable outputs and outcomes [12]. Although medical AI is a relatively new field, it is important to acknowledge that the underlying disparities in healthcare that drive bias in medical AI are not recent developments. Rather, they are rooted in longstanding historical driving forces of inequality in health systems, which themselves reflect even longer-standing discrimination and other forms of structural oppression [11,38].

Below, we discuss potential biases that can arise at different stages in the AI development pipeline, including biases in training data, model development, model implementation, and publication. At each stage, we first discuss the nature of these biases and how they can affect AI models and downstream clinical decision-making. We then highlight illustrative real-world and hypothetical examples that were intended (or were implemented) for aiding clinical decisions. Finally, we provide a thorough discussion of potential solutions and techniques that may or may not mitigate each bias. Each of these techniques has its own advantages and limitations, and the choice of method should be guided by the specific characteristics of the data set and the clinical task at hand. Table 1 summarizes the potential clinical consequences and mitigation techniques for the biases and bias-related issue presented throughout this narrative review.

**Table 1:** Types of bias through medical AI development stages, where they occur, their potential clinical consequences, and mitigation techniques.

Medical AI development stage	Bias/issue	Illustrative example	Potential clinical consequences	Bias mitigation strategies
Training data	Imbalanced sample sizes	Training data sets often overrepresent non-Hispanic Caucasian patients	Worse performance and algorithm underestimation for underrepresented groups	- Characterize data set patient sociodemographics - Methods for imbalanced data (oversampling, data augmentation) - Cultivation of large, diverse data sets
	Nonrandomly missing patient data	Low socioeconomic patients often have more missing data in EHR	AI model systematically underestimates risks or misses important factors for certain patients	- Imputation techniques - Improved data collection methods - Record linkage algorithms
	Data not usually or easily captured	Social Determinants of Health (SDoH)	AI model makes less accurate predictions for patients whose health is significantly impacted by SDoHs	- Standardized questionnaires, surveys - NLP/LLM methods for unstructured clinical text - Incorporating external, public data
	Biases in data labels and misclassification	Provider cognitive biases	Perpetuation and amplification of existing biases and healthcare disparities	- Expert consensus labeling - Cultural competency training
	Race and ethnicity in clinical algorithms	Race/ethnicity correction factors in risk calculators	Race/ethnicity is not a biological construct	- Socioeconomic deprivation factors can be more representative - Use of zip codes as a proxy for SES
Model development and evaluation	Overreliance on whole-cohort performance metrics	Only evaluate and optimize whole-cohort accuracy or AUC	Model learns good predictions for well-represented patient groups, but performs much worse in underrepresented patient groups	- Subgroup analysis - Bias-centered optimization metrics - Explicit statistical debiasing methods - Model interpretability methods
Publication	Where medical AI models are developed and published, and by whom	Over 50% of published clinical AI models use data from US or China	Overrepresentation of certain priorities, viewpoints, incentives	- Data collection from multiple countries, healthcare systems - International data sharing initiatives
	AI research is biased towards positive results and certain medical domains	Radiology papers accounted for over 40% of AI publications in 2019	Lack of published negative results provides incomplete view of clinical AI limitations	- Multidisciplinary collaboration of developers and clinicians - Journals and funding agencies incentivize publication in underrepresented medical specialties and negative results
Model implementation	Sample selection bias: real-world patients differ from training cohort	Epic sepsis model	Differential deterioration of model’s performance and clinical utility across patient groups	- Ongoing monitoring systems that detect and quantify bias in model predictions - Explicit regulations for reporting bias and demonstrating fairness - Clinical trials for AI validation
	End user biases	Convoluted user interfaces, documentation burdens, inherent physician mistrust	Physicians follow AI recommendations for certain patients but override it for others	- Infrastructure for continuous quantitative and qualitative feedback - Regular retraining and model updates - Developers work with hospital leaders and care providers to align model with clinical workflow - Interpretable AI for clinician trust and adoption

## Biases in training data

The first step in developing medical AI models involves collecting and preparing data. Biases in the data used to train these models can appear in several forms, some of which are more overt than others.

### Imbalanced sample sizes

#### Definition and clinical consequences

Perhaps the most straightforward form of data bias is the presence of imbalanced (insufficient) sample sizes for certain patient groups. Many data sets used to train AI models for clinical tasks overrepresent non-Hispanic Caucasian patients relative to the general population [39], and more broadly, over half of all published clinical AI models leverage data sets from either the United States or China [40]. When an algorithm is trained on imbalanced data, this can lead to worse performance and algorithm underestimation for underrepresented groups. An underestimating algorithm will forgo informative predictions in underrepresented groups—especially on outlier cases—in favor of approximating mean trends in order to avoid overfitting [41]. Without statistically (or clinically) meaningful predictions for certain groups, any downstream clinical benefits of the AI model are limited to only the (largest) groups with sufficient data sizes [13]. The benefits and clinical improvements that result would be similarly constrained, perpetuating healthcare disparities. Imbalanced samples are a form of bias that the medical community has historically and still currently struggles to address [42].

#### Illustrative examples

One illustrative example of class imbalance affecting model performance is the prediction of melanoma from skin images. Existing disparities in (non-AI) melanoma diagnoses are well-documented: at the time of diagnosis, darker-skinned patients already present with later stages of the disease and have lower survival rates than fair-skinned patients [43]. Early diagnosis of melanoma is key to effective treatment, and thus AI-based melanoma prediction has great potential for clinical use [43]. Unfortunately, disparities in AI-based melanoma prediction models are common: the majority of these models are trained on data sets (such as the Melanoma Project) that are heavily composed of light-skinned images from patients in the US, Europe, and Australia (imbalanced sample bias) [43]. These models exhibit worse performance for images of lesions in darker skin tones, which resulted in worse predictivity when deployed in real-world settings [43]. Although melanoma incidence occurs more frequently in non-Hispanic white individuals and is phenotypically different on dark skin, this should not justify the exclusion of these patient groups from the benefits of AI-based melanoma detection.

Another noteworthy study aimed to systematically quantify the effect of class imbalance on the fairness and generalizability of an AI model across different patient groups [44]. The model of interest was trained on the MIMIC-III data set (an open source, real-world ICU data set) to predict in-hospital mortality [44,45]. The researchers found that imbalanced representation of racial groups had a significant effect on model performance (yielding recall rates as low as 25%), raising critical concerns for the model’s potential application in real clinical settings [44].

#### Mitigation strategies

Given the frequency and impact of imbalanced data bias, AI developers can proactively counteract its effects on models and downstream clinical decision-making. Prior to any model development, a helpful first pass would be to review and characterize the data set of interest, ensuring appropriate representation across racial, ethnic, and other sociodemographic dimensions.

During data preprocessing, statistical methods that account for data imbalance can be employed. A popular strategy is oversampling, which aims to balance the data set by increasing the number of instances in a minority class that a model is trained on [46–48]. Common techniques include Synthetic Minority Over-sampling Technique (SMOTE), which generates synthetic examples for the minority class by interpolating between existing minority class instances, and Adaptive Synthetic Sampling (ADASYN), which uses a similar method to SMOTE but focuses more on examples that are harder to classify (near the decision boundary) [49,50]. Another related strategy is data augmentation, which involves generating new samples based on random perturbations of existing data points [51–53].

Standardized reporting checklists such as the Prediction Model Risk of Bias Assessment (PROBAST) tool have been developed to assess the risk of imbalanced data biases, which allows both developers and downstream users (e.g., physicians, hospitals) to better understand the suitability and limitations of AI-based systems in specific clinical settings [54–56].

Ultimately, the most powerful solution for sample size biases is to cultivate large, diverse data sets. Although a resource-demanding process, such data sets can successfully represent and account for variations within and across patient groups and will most directly lead to equitable and generalizable AI for clinical decisions. Collaborative efforts across institutions and countries can facilitate the creation of such representative data sets.

### Capturable but missing data

#### Definition and clinical consequences

AI models are computationally superhuman in that they can quickly process and incorporate massive amounts of data in parallel, e.g., thousands of past and present lab values in a patient’s electronic health record (EHR). On the other hand, a key limitation of these algorithms is that they can only use data that is readily available, and these data can be missing nonrandomly. This is especially true when models are applied to clinical use-cases: Patients with low socioeconomic status (SES) have been shown to receive fewer diagnostic tests and medications for certain (chronic) diseases [57]. These patients are also more likely to receive care at multiple health institutions, which may utilize different EHR systems (e.g., Epic versus Cerner) for storing patient data [57]. The recent advance of telehealth has greatly increased the availability of patient-reported data collection (e.g., pain surveys on smartphone apps) [58], but certain patient groups may have lower (digital) health literacy or may be less able to self-report health outcomes. Even a patient’s choice to seek care at all varies across sociodemographic factors [59]. Inequities in data missingness carry over to AI tools trained on these data, especially those developed for assisting in clinical decision-making. Such AI systems could systematically underestimate risks or miss important factors for these patients, leading to worse care recommendations.

#### Illustrative example: ED bed allocation

For example, an algorithm that aims to identify high-risk patients admitted to the emergency department for priority bed allocation might incorporate past medical history into its risk computation, such as the presence or frequency of certain ICD codes (previous diagnoses or procedures) from the patient’s existing EHR data (e.g., past heart failure). From the algorithm’s perspective, the data either contains or does not contain the ICD code; the algorithm cannot discern patients who “truly” have not had a prior heart failure (despite a robust EHR record) from patients who only do not have the ICD record due to some other factor (stored in the EHR of another institution). In the latter case, the algorithm would assign a lower risk for these patients, which would systematically bias them toward a lower risk prediction and may fail to qualify them for priority bed allocation. Thus, AI models trained on non-randomly missing patient data can yield worse clinical utility for certain patient populations.

#### Mitigation strategies

Several mitigation strategies can be implemented to address the issue of capturable but missing data. One common solution involves removing patients with a certain threshold of missing variables from the training set. However, selecting for only near-complete data can remove large portions of certain populations, biasing the data further and leading to uninformative predictions for those groups. A better alternative involves statistical techniques such as data imputation, where missing variables are filled in with likely values based on similar patients (e.g., regression or nearest-neighbor based multiple imputation), which can help mitigate bias caused by differential missing data [60,61]. Engineering composite features that are less sensitive to individual missing data points (trend-based variables such as slopes) have shown resilience to data gaps [62].

Regarding fractured care across multiple EHR systems, strengthening data sharing protocols and improving interoperability between health record systems can help ensure completion and continuity of patient data for AI model training. Initiatives such as the Fast Healthcare Interoperability Resources (FHIR) standard will be critical towards this goal [63]. Probabilistic record linkage algorithms can help facilitate the combination of patient records across health systems and data sets, reducing gaps in missing patient data [64,65]. Increased efforts have been made to improve health literacy and encourage more consistent healthcare engagement across diverse demographic groups [66,67], and in parallel, user-friendly interfaces for patient-reported outcome measures, accommodating various levels of digital literacy, have shown promise in increasing data completeness [68,69]. Developing protocols for targeted follow-up with patients may assist the collection of high-value missing information, and design user interfaces that highlight missing data to clinicians may encourage more complete data entry during patient encounters.

### Data that are not usually captured: Social determinants of health

#### Definition and clinical consequences

Social determinants of health (SDoH) are an illustrative example of data that are not often explicitly captured in patient records. SDoHs can be dually defined as both (1) the social factors that promote or undermine individual or population health; and (2) the social processes that underlie the unequal distribution of these factors across groups [70,71]. Examples of SDoHs include access to care, social support networks, education, transportation, and clinical knowledge [71,72]. These determinants can have profound effects on patients’ health, particularly on older patients that have disproportionately accumulated these effects over the life course (the “Weathering” effect) [73–75]. While some factors like access to care can be proxied (e.g., using zip codes), others such as social supports are likely not explicitly captured in patient records or are only sparsely present in unstructured form (e.g., clinical notes).

Incorporating such data is challenging, often requiring the balancing of competing priorities and incentives from multiple stakeholders. EHR software companies must invest time and finances into developing necessary data infrastructures and user interfaces. Full research studies may be required to create meaningful and approved operationalizations of SDoHs. Even then, the burden of parsing SDoH information that is clinically relevant (e.g., from verbally or anecdotally conveyed information) may lie on a provider who is overwhelmed with administrative duties. Nevertheless, incorporating SDoHs and similarly uncaptured data is crucial toward building clinically useful and unbiased decision-making tools. Failure to do so may lead not only to worse performing models overall (missing out on clinically meaningful information) but also disproportionately worse performance in groups whose SDoHs have increased and compounded effects [76]. In turn, a deployed AI system that does not incorporate SDoH data may produce worse care recommendations for patients whose health is significantly impacted by social determinants.

#### Mitigation strategies

Standardized, easy-to-use data instruments like structured questionnaires or screening surveys that can (1) quantify SDoHs; and (2) be easily incorporated into EHRs and clinical workflows would help facilitate systematic collection of SDoH data. Some studies have already shown promise in both the logistical feasibility and clinical utility of these instruments [77]. More recent natural language processing (NLP)-based methods, particularly LLMs like GPT-4, have also shown success in capturing SDoH information from unstructured clinical notes and patient narratives, yielding tangible benefits for patient outcomes [78,79].

Incorporating external, publicly available data sets on individual and community-level SDoH factors, such as census data and environmental quality indices, can provide valuable context to individual patient data about factors like living conditions and neighborhood resources. Developing secure data-sharing partnerships with social service agencies and community organizations to access relevant SDoH information can further enrich the collection of these information. Government programs and funding (reimbursement) initiatives can provide a system-level solution by incentivizing healthcare organizations to capture SDoH data. Better capturing of these SDoHs may in turn more equitable and effective policymaking, especially through policies like Medicare and Medicaid that enable greater healthcare access for marginalized populations [80,81].

### Data labels and misclassification

#### Definition and clinical consequences

A third form of data bias involves the labels that supervised machine learning models are trained to predict. Examples of common prediction targets include in-hospital mortality, 30-day unplanned readmission, length of stay (LOS), and diagnosis. The same implications of nonrandom missing patient data described previously (related to patient features) also apply to prediction labels. However, additional factors that drive bias in labels must be considered, along with their consequences for clinical decision-making. For the purposes of a supervised training paradigm, data labels are considered the “ground truth” from which models are optimized [82]. However, in clinical settings, the outcome (e.g., diagnosis) reflects a subjective decision made by a single care provider.

A natural consequence of data label bias emerges in the form of misclassification, which can be defined as systematic errors that occur when individuals are assigned to a category other than the one they “should be” assigned to [83]. In clinical settings, what exactly constitutes a misclassification can be difficult to define. A relatively clear misclassification could involve the assignment of a hypertension diagnosis despite normal-range blood pressure values [83]. Whether that patient was “correctly” discharged shortly afterwards, however, may be less objective. Furthermore, the degree of practitioner misclassification can vary along sociodemographic factors. This can be partially attributed to disparities in healthcare systems and the delivery of medical care: Low SES patients are more likely to be seen at teaching institutions, where clinical reasoning may be systematically different than other clinical settings serving higher SES individuals [84]. Furthermore, uninsured and publicly insured patients (regardless of institution) receive worse medical care than those with private insurance [85]. Implicit cognitive biases of healthcare providers related to patient attributes (gender, race) can also lead to variation in diagnoses (misclassification rates) and the quality of care, manifesting anywhere from body language and word choice to biased treatment decisions [84]. Consequently, AI models trained on potentially biased labels may perpetuate and amplify not only differential misclassifications and substandard care practices based on these social factors, but also the original cognitive biases in its own predictions and recommendations.

#### Mitigation strategies

One solution for mitigating misclassification involves obtaining higher quality labels through expert consensus. Multiple physicians can provide independent labels (e.g., diagnosis) can help reduce individual bias. This approach can be particularly effective when combined with methods to assess inter-rater reliability and resolve discrepancies. When appropriate, ensuring diversity of expertise and background in data labeling teams can help mitigate systematic biases. Still, this process is expensive, time consuming, and may not fully remove the effects of differential misclassification. Quantification of label and prediction uncertainty provides another potential solution, where Bayesian approaches and ensemble methods can be employed to provide confidence intervals or probability distributions of predictions [86–88]. Beyond the AI development pipeline, more direct healthcare initiatives, such as implicit bias and cultural competency training programs, can improve awareness of unconscious biases that may affect clinical decision-making. Reducing provider bias during diagnostic and treatment decisions has been shown to demonstrably improve both algorithm performance and overall clinical care quality [89].

### Race and ethnicity in clinical algorithms

#### Definition and clinical consequences

In addition to racial and ethnic imbalances in training data, there has been increased scrutiny on the use of race and ethnicity in predictive clinical algorithms [9]. These concerns are rooted in a few factors. Race and ethnicity are social constructs, not biological constructs [9,90]. Indeed, there is mounting evidence that race is not a reliable proxy for genetic differences and, despite this lack of evidence, it has become acceptable to adjust for race even without understanding what it represents in a given context [9]. The meaning of ethnicity can be context dependent, ranging from national origin to categories that governments use to collect data for administrate and other purposes [90]. Another related factor is overreliance of association without proper evidence of causation: Racial differences may only be surrogates for socioeconomic or cultural deprivation [91]. The point is not to neglect the fact that, as societal constructs, race and ethnicity can influence health and disease, but rather to be as precise as possible about the causative social (e.g., racism, social class) and biological factors (e.g., genetic variation) [90]. This is of particular importance when using AI methods that typically lack straightforward interpretability and that prioritize predictive performance over mechanistic understanding. Third, attribution of race and ethnicity by healthcare providers to patients is demonstrably unreliable [90], further complicating the use of these factors in AI models. There are several well-documented instances of insufficient evidence for race and ethnicity to be used as predictive factors in models. For example, there is limited evidence for using black race as a factor for calculating estimated glomerular filtration rate, a critical measure of kidney function [92]. Using race as a factor leads to overestimation of kidney function in black patients, potentially leading to longer times to get on kidney transplant lists [92–95].

#### Mitigation strategies

To extend beyond (or even replace) race as an input feature for medical AI models, there have been significant efforts to reassess the use of race in risk calculators, which often leads to its elimination as a factor. Recent examples include cardiovascular risk assessment [96], kidney function (e.g., estimated glomerular filtration rate) [93–95], and risk of urinary tract infection in infants [97,98].The American Heart Association’s PREVENT Equations use a social deprivation index to recognize the impact that social deprivation can have on cardiovascular-kidney-metabolic conditions [96,99]. The Organ Procurement and Transplantation Network recently required kidney programs to assess their waiting lists and correct waiting times for any black kidney candidates disadvantaged by the overestimation of their kidney function due to race-inclusive calculations [95]. The development of optimal calculators for estimating risk of urinary tract infection in infants is ongoing and reflects the complex interplay between social constructs, clinical evidence, data analytics, and clinical change management [98]. Greater capture and utilization of social determinants of health in medical AI models for clinical risk prediction will be paramount.

### Bias in model development and evaluation

#### Definition and clinical consequences

The next stage of developing a clinical AI model involves the selection and training of algorithms (model development) and the subsequent evaluation of these models on independent patient cohorts [11]. These steps inherit biases in the training data and can also introduce their own biases. A naïve approach to developing medical AI models on the part of the developer would be to assume inherent objectivity in model outputs. From the model’s perspective—and perhaps the developer’s as well—the best-performing solution is the one with the lowest loss or the best performance metric (e.g., area under the curve; AUC). But this may result in a model learning good predictions for well-represented patient groups but performing much worse in underrepresented patient groups. Model evaluation is thus a crucial checkpoint at which developers can identify bias introduced during model training.

#### Mitigation strategies

One way to identify bias introduced during model development is the method of subgroup analysis [100]. In addition to the overall performance, the model developer will evaluate and report their AI model’s performance across patient subgroups (e.g., for Low versus High SES patients, public versus private insured patients). Alternatively, or in parallel, a developer might choose to leverage alternative optimization metrics beyond accuracy or AUC that quantify bias (and thus inherently aim to debias model performance). Such quantitative bias metrics include equalized odds and predictive parity [101]. Importantly, these bias metrics should be chosen based on the algorithm’s intended clinical use. For example, a model that exhibits predictive parity (no difference in precision across groups) may not have equalized odds (similar sensitivity and specificity across groups), which metric is more important to optimize on depends on the intended clinical use-case [101].

Model interpretability is another important step in both validation and bias mitigation, offering insights into how decisions are generated (e.g., which features are most involved) or explaining the behavior of “black box” models (e.g., deep learning models with millions of parameters) [11]. Understanding how an AI model leverages features to make predictions allows validation against current standards of care. Interpretability can also detect bias by examining how the features that drive model predictions differ for patients in different subgroups (which may be differentially aligned with best care practices) [101]. Popular interpretability methods for machine learning models include Shapley Additive Explanations (SHAP) and Local Interpretable Model-agnostic Explanations (LIME) [102,103].

During model training, the loss function can be modified to assign higher weights to samples from underrepresented groups, encouraging the model to prioritize correct predictions for these samples [104]. Regularization terms that penalize disparities in prediction across subgroups can achieve a similar result. Other statistical debiasing approaches for AI algorithms that rigorously employ models into accounting for underrepresented groups include adversarial debiasing and Prejudice Regularization [105,106]. These methods have been successfully shown to achieve equitable subgroup performance while maintaining or even improving overall model performance [107,108], demonstrating that bias mitigation need not be at the expense of overall performance.

### Bias in publication

#### Definition and clinical consequences

Where models are developed and published can yield additional sources of bias in medical AI solutions for clinical decisions. One study found that over half of all published clinical AI models in 2019 used training data from the US or China, which likely reflects the advanced technological infrastructure of these countries conducive to AI development [40]. This study also demonstrated disparities in author’s gender (75% male), expertise (authors were predominately “data experts” rather than clinical experts), and medical domain (radiology was substantially overrepresented at over 40%) [40]. Together, these factors can bias the trajectories and priorities of future medical AI publications: a data scientist may have different goals than a clinician with respect to model development and implementation (e.g., optimizing performance versus maximizing clinical utility). This could lead to an overemphasis on developing AI for certain applications while neglecting other that may be equally or more impactful in clinical settings. Furthermore, the tendency of journals to favor AI studies with positive results—especially those that demonstrate superior performance—skews the published literature, giving an incomplete view of the AI landscape [109]. Benchmark data sets are commonly used to demonstrate relative (superior) performance, and only algorithms that do well on these benchmarks get published [110].

#### Mitigation strategies

Researchers should strive to include data from a wider range of countries and healthcare systems. This could involve international collaborations and cross-country data sharing initiatives, facilitating AI model training on diverse and more globally representative data sets and thereby improving their clinical utility for more patient populations. To reduce biases introduced by domain-specific perspectives, AI research teams should consist of collaborations between clinicians, who understand practical healthcare needs, and data scientists, who specialize in model development, which can lead to models that prioritize clinical utility over solely technical performance. Journals and funding agencies might encourage and incentivize multidisciplinary co-authorship for studies that develop AI solutions intended for clinical use, particularly in underrepresented medical AI domains like primary care, mental health, and pediatrics [40]. To counteract positive-result publication bias, journals might create dedicated spaces for publishing negative results or unsuccessful AI models, providing valuable insights for the field. In parallel, journals might require more detailed failure commentary, where authors describe where and why their models may not perform well in certain clinical contexts. Implementing preregistration of AI development for clinical use where studies must be evaluated on methodology (regardless of final outcome) may also reduce publication bias.

## Bias in model implementation

The integration of AI models into real-world clinical workflows involves several stakeholders across multiple levels of a healthcare system, including hospitals, providers, data scientists, software engineers, and the government. Critically, bias can still emerge at this stage, even in models that have received regulatory approval or appear to have “fully debiased” outputs.

### Model deterioration in the real world: Sample selection bias

If the real-world patient cohort of interest differs from patients in the training and/or validation data set (e.g., by demographic makeup), medical AI model performance can deteriorate and can do so differentially for different patient groups [11]. A similar trend can occur when models trained at one institution are applied to the data at another institution [13]. This phenomenon, known as sample selection bias, is well-studied in the literature and can lead to biased and potentially harmful decisions in real-world clinical settings [111].

A well-known example of sample selection bias is the Epic Sepsis Model (ESM), developed on EHR data to generate automated warnings for clinicians that patients may be developing sepsis [112]. The study authors found that the ESM had significantly worse AUC, sensitivity, and specificity after deployment relative to initial reports, missing two-thirds of sepsis cases and frequently issuing false alarms [112]. The fact the ESM performed much worse in real-world deployment than in initial reports highlights the risk of AI systems being used clinically before their real-world performance across diverse populations is thoroughly validated.

Deployed AI models for clinical decisions like the ESM can inappropriately increase triage and trigger unnecessary diagnostic testing and prescriptions, decreasing the quality of clinical care and increasing hospital costs [101]. A key strategy toward mitigating bias at the deployment stage, therefore, involves proper feedback loops that can continuously monitor and verify model outputs and performance in clinical settings, particularly how these measures differ across sociodemographic factors. Ongoing monitoring systems should also detect and quantify biases in model predictions using bias metrics discussed previously. In addition to the techniques for mitigating data biases discussed previously, advanced statistical methods that aim to address sample selection bias by target population identification are under development [111].

### End users of AI models

Another potential source of bias in model implementation is how end users (e.g., physicians) use or do not use the deployed model solution. Initially, a physician may hesitate to trust a model without understanding its underlying reasoning, which may prevent the realization of equitable outcomes; model interpretability methods can help resolve this issue. Furthermore, some clinicians may also choose to use or ignore model recommendations for other reasons such as convoluted user interfaces or documentation burden, potentially leading to bias in when AI is applied. If doctors are more likely to follow AI advice for certain patient groups but override it for others, this could lead to inequitable application of the AI system. Feedback infrastructures like surveys that can capture why these behaviors occur must therefore be implemented to help understand and account for these factors. Developers must work in conjunction with hospital leaders and care providers to align model implementation with clinical workflow demands [101]. Integration of dashboards, visualizations, or notifications that help clinicians understand potential biases in AI-assisted decisions may facilitate physician awareness of AI limitations.

### Validating AI for unbiased clinical application

The FDA’s Software as a Medical Device (SaMD) Action Plan regulates the translation of AI models into real-world clinical use, often dubbed “translational AI” [10]. However, while the evaluation criteria of SaMD focus on risk mitigation and the reproducibility and robustness from the software perspective, there are no explicit FDA regulations that examine fairness or bias in medical AI outputs, and indeed, many AI-driven SaMDs have displayed substandard performance among racial and ethnic minorities [113]. Already, AI is employed in clinical trials to accelerate enrollment, monitor patients (e.g., wearables), and improve retention [29,114,115]. Prior to real-world implementation in clinical settings, we argue that rigorous validation of translational AI is through clinical trials: AI should not only meet criteria for performance, usability, and safety risks, but also report and demonstrate unbiased application.

## Biases in medical large language models

Although recent LLMs have achieved unprecedented performance on NLP tasks in medicine and other domains [116–118], medical LLMs deployed for clinical decision-making pose unique limitations and risks with respect to bias [119–122]. LLMs are autoregressive in nature, which means they rely on the statistical patterns of which words have preceded others in their training text corpora [123]. If a medical LLM has been trained on data that contains misinformation or biased content, LLMs deployed for clinical decision-making are at risk of reproducing these problematic contents [120,124–126]. This could lead to the AI system generating biased and inaccurate information to clinicians. Furthermore, an LLM cannot assess itself—it does not know whether the clinical guidelines it produces are accurate or harmful, recent or outdated [120]. Finally, due to the probabilistic nature of LLM output generation, LLMs can produce different outputs despite being prompted by the same instructions multiple times. In a clinical setting, this inconsistency could lead to unreliable or inconsistent recommendations, potentially affecting patient care. The bias mitigation techniques discussed throughout this review apply to medical LLMs, particularly proper mechanisms for filtering and clinical verification of training data, as well as continuous physician oversight and awareness of an LLM’s potential to “hallucinate.” Some statistical debiasing methods designed for language models are emerging; however, their implementation in real-world clinical LLMs remains limited [127–132].

## Conclusion

Bias can occur at all stages in the medical AI development pipeline, including biases in data features (imbalanced samples, missing, or hard to capture variables), data annotations (implicit provider biases), model development and evaluation (developer naivety), model implementation (real world generalizability, end-user acceptance and utilization), and publication (authors and their priorities). These biases can have detrimental effects on both models and clinical decision-making, which can contribute to and exacerbate longstanding disparities in healthcare.

Addressing these biases is becoming increasingly possible due to methods and metrics that can uncover and mitigate bias, but more research is needed. Going forward, larger and more powerful medical AI models will be increasingly data hungry; efforts to collect larger and more representative patient data must parallel these advancements. Improved bias reporting and transparency in literature will be critical, as will the development of methods tailored to detecting and mitigating bias in clinical decision-based AI. Additional discourse on intersecting sources of bias that affect AI and clinical decision-making will be paramount, including biases in healthcare regulation and insurance. Debiasing medical AI models will prove crucial in preventing the perpetuation and exacerbation of health disparities and ensuring all patients benefit equally from the future of medical AI.
